# An ecosystem of interconnected technologies to increase efficiencies in blood establishments: The example of the Blood and Tissue Bank of Aragón, Spain

**DOI:** 10.1111/vox.13752

**Published:** 2024-10-23

**Authors:** Ana Isabel Pérez Aliaga, Irene Ayerra, Marcia Cardoso, Fernando Puente, Alfonso Aranda, José María Domingo, Rosa Plantagenet‐Whyte

**Affiliations:** ^1^ Blood and Tissues Bank of Aragón (BTBA) Zaragoza Spain; ^2^ Hemotic Olite Spain; ^3^ Terumo Blood and Cell Technologies Zaventem Belgium

**Keywords:** blood banking improvement, pathogen reduction technology, production algorithm, productivity, traceability, whole‐blood processing

## Abstract

**Background and Objectives:**

Blood establishments face environmental, financial, demographic and societal challenges that may impair sustainable blood supply to patients. This study presents the technologies (devices and software) assembled in a global ecosystem implemented by the Blood and Tissue Bank of Aragón (BTBA), Spain, over the last decade to overcome these challenges.

**Materials and Methods:**

Descriptive yearly activity data (2013–2023) of BTBA were retrospectively collected to evaluate the impact of different technologies on blood processing efficiency, focusing on the production of blood components (red blood cell concentrates [RCCs], platelet concentrates [PCs]) and plasma. Operator satisfaction about the technologies introduced in daily routine work was also monitored.

**Results:**

Between 2013 and 2023, the annual production decreased by 16.0% for RCCs and increased by 13.3% for PCs. From 2020, all PCs were treated with pathogen reduction technology, and no inventory stock‐out was reported. The lowest PC expiry rate (0.2%) was observed after the implementation of the software for blood processing and PC stock management. The deployment of this software also improved plasma recovery: on average, an extra plasma volume of 9 mL was collected per donation in 2023 compared to 2015. A survey confirmed staff satisfaction.

**Conclusion:**

The progressive implementation of automated and software‐based solutions was key to increasing efficiencies in BTBA. This enabled the optimization of blood processing by maximizing productivity, enhancing traceability, reducing overproduction and wastage and increasing the yield of recovered plasma, while ensuring blood product safety and staff satisfaction.

**Highlights**

- The Blood and Tissue Bank of Aragón (BTBA), Spain, has implemented an ecosystem of interconnected technologies to overcome challenges that potentially impair the sustainability of blood supply.
- This ecosystem involves a fully automated blood processing system combined with pathogen reduction technology, supported by software‐based decision making and data connectivity.
- A descriptive review of the BTBA activity data from 2013 to 2023 shows that this ecosystem was instrumental in optimizing blood processing, maintaining safety of blood components and ensuring staff satisfaction.

## INTRODUCTION

Blood transfusion is one of the most frequent medical procedures and involves a series of perfectly linked activities allowing the patients to receive suitable blood components on time and in accordance with the highest quality and safety standards.

Blood establishments (BEs) are currently facing environmental, financial, demographic and societal challenges, which, if not addressed, could jeopardize the sustainability of transfusion services. Among these challenges, two major contributors affecting the balance between blood demand and supply are the ageing of the blood donor population (with some donors becoming patients) [1] and the difficulty in retaining young donors [2]. Moreover, while the demand for red blood cell concentrates (RCCs) has gradually decreased over the past decade as a result of improved patient blood management, the demand for platelet concentrates (PCs) and plasma‐derived medicinal products has steadily increased [3, 4, 5, 6]. Maintaining the safety of blood transfusions is also a constant challenge in light of globalization [7] and climate change [8]. This requires not only the implementation of standard reactive measures for donor selection and screening but also proactive approaches, such as pathogen reduction technologies (PRTs).

In addition, BEs are bound by standardization and documentation requirements issued by regulatory agencies such as the European Directorate for the Quality of Medicines & HealthCare, necessitating quality management systems that ensure complete traceability of blood products from collection to processing, storage and transfusion [9]. At the same time, BEs face logistical challenges in human resource management, including high staff turnover which creates a continuous need for training and diversification of operations to keep employees engaged. Many BEs are addressing this issue by widening their activities, such as establishing tissue and organ banks and expanding into the fast‐growing market of cellular therapy. These new activities bring additional complexity to blood management, including a need for additional space. However, this can be achieved through increased automation in blood processing labs [10]. Furthermore, BEs must strive to maintain operational efficiency while exploring and developing new treatment opportunities that contribute to a sustainable health system without significantly increasing costs, or ideally reaching cost neutrality.

The Blood and Tissue Bank of Aragón (BTBA) is a public service located in the region of Aragón (Spain), which is home to more than 1,300,000 inhabitants [11]. In 2023, this BE collected 40,136 whole‐blood (WB) donations and supplied 6392 PCs to 19 transfusion services (12 public and 7 private hospitals), 4 of which are also BTBA collection sites.

The objective of this study was to present the various approaches and technologies that BTBA has gradually implemented over the last decade. These efforts were aimed to create an ecosystem of automation and software‐based decision making to improve operational efficiencies and address previously identified challenges.

The study specifically focused on conducting a comprehensive review of BTBA's activity data following the introduction of Reveos® technology in 2013. Additionally, the study examined the outcomes of subsequent technological implementations, such as PRT and LHEMA software, introduced between 2015 and 2023. By analysing activity data from 2015 to 2023, the study aimed to identify the impact of these integrated technologies on blood processing optimization and to assess the level of staff satisfaction associated with these changes.

## MATERIALS AND METHODS

### The Reveos® system and the T‐Pool Select tool

BTBA has been routinely using the Reveos Automated Blood Processing System (Terumo Blood and Cell Technologies [BCT]) since July 2013 [10]. The design and functioning of this device have been extensively described in several publications [10, 12, 13]. Briefly, the Reveos system is able to process up to four WB donations during a single centrifugation run of 20 min into two (plasma and RCCs; 2C separation protocols) or three blood components (plasma, RCCs and platelets; 3C separation protocols) [12, 14]. The Reveos system includes a software solution (T‐Pool Select) to select interim platelet units (IPUs) to be combined in the production of the required number of PCs with a minimum yield [15], in accordance with internal, national and international specifications [16]. Data regularly collected as part of quality control procedures showed that automation in blood processing improved the quality of manufactured blood components in comparison with the semi‐automated buffy coat method previously used in this BE [10].

### Pathogen reduction technology

BTBA implemented the Mirasol® Pathogen Reduction Technology (PRT) System (Terumo BCT) in late 2015 to improve the safety of platelet transfusions [10, 16, 17]. Treatment with PRT allows the extension of the shelf‐life of PC from 5 to 7 days at standard storage conditions [9, 10, 18].

### Cryoconserved PCs

Platelet cryopreservation following a modified Valeri method [19] was initiated in 2016 to decrease PC expiry rates while creating an emergency stock to meet urgent demands for PCs in remote hospitals [19, 20].

### Software‐based decision making and data connectivity

The LHEMA software (Hemotic) was implemented on a routine basis in the BTBA in April 2022. This standalone software, which is not connected to the blood bank information system, requires minimal manual input for data entry (eight parameters must be specified) before generating production recommendations for blood components adjusted to the forecasted demand. Additionally, it is capable of alerting the user to potential blood shortages, allowing for the necessary adjustments to be made in advance to meet the demand for PCs by 1 week. Furthermore, it provides precise information regarding the number of additional WB donations required to avoid such situations [21].

BTBA started using the Terumo Operational Medical Equipment Software (TOMEs; Terumo BCT) in April 2021 to ensure a better oversight of the pooling process through seamless data transmission and monitoring of connectivity among interoperable Terumo BCT systems (Reveos, Mirasol illuminator devices, T‐Pool Select, TSCD‐II® sterile connectors).

### BTBA activity data

As an extension to the study from Pérez Aliaga et al. [10] covering the 2007–2019 activity period, retrospective BTBA activity data between January 2013 and December 2023 were collected to evaluate the impact of the different technologies implemented on blood processing efficiency. These descriptive data include the numbers of WB donations processed (overall and per‐separation protocol) and discarded, RCCs processed and discarded, IPUs discarded, PCs processed (including those treated with Mirasol PRT) and discarded, PCs expired (in the BTBA, hospitals or Aragón transfusion network, i.e., the joint network of BTBA and hospitals), PC stock‐out events, the remaining shelf‐life of PCs upon delivery to transfusion services and the volume of plasma recovered from WB units.

### Monitoring of staff satisfaction

Staff satisfaction was monitored by collecting operator feedback at two time points. In 2013, the staff (*N* = 13) was asked to rate the usability aspects of the newly implemented Reveos technology in comparison with the formerly used buffy coat method on a scale from 1 (*lowest usability score*) to 5 (*highest usability score*). In 2023, the operators working at BTBA (*N* = 18, of whom 7 participated in the 2013 satisfaction survey) were asked about the new technologies that had been implemented over the last decade (Reveos system, T‐Pool Select program, LHEMA software, TOMEs middleware), using the same grading scale. They were also asked to choose what they considered as the most important added value of any new technology or equipment for their routine work among four possible answers (ergonomics of workstation and operator's safety, error minimization, optimization of blood processing, workload reduction). In addition, operators who participated in the survey could only respond to questions about technologies with which they had first‐hand experience (e.g., an operator who did not work before the TOMEs implementation could not answer questions on comparisons with the way of working without this software).

## RESULTS

### WB donations, RCC production and plasma recovery

The annual number of WB donations collected by BTBA decreased by 16.0% between 2013 (47,782 units) and 2023 (40,136 units). Following this, the annual production of RCCs decreased by 15.9% between 2013 (46,187 units) and 2023 (38,826 units); statistics from the years 2015 to 2023 are presented in Table 1.

**TABLE 1:** Evolution of Blood and Tissue Bank of Aragón (BTBA) activity data in relation with the technologies implemented.^a^

	2015	2019	2020	2021	2022	2023
	13% PRT	60% PRT	90% PRT	Algorithm validation	LHEMA software	Automation of protocol selection and improvements to the LHEMA software
WB processing						
Total WB donations processed with Reveos system, *n*	42,454	41,165	39,427	41,187	40,260	39,621
Discarded WB donations, %	0.10	0.02	0.04	0.04	0.05	0.07
WB donations separated with 2C protocols, *n* (%)	1956 (4.6)	2479 (6.0)	3738 (9.5)	7064 (17.2)	6212 (15.4)	9583 (24.2)
2C overnight protocol, *n*	1058	1358	3034	6097	4822	8135
2C fresh protocol, *n*	898	1121	704	967	1390	1448
WB donations separated with 3C protocols, *n* (%)	40,498 (95.4)	38,686 (94.0)	35,689 (90.5)	34,123 (82.8)	34,048 (84.6)	30,038 (75.8)
3C overnight protocol, *n*	15,450	23,011	22,257	18,229	16,731	27,772
3C fresh protocol, *n*	25,048	15,675	13,432	15,894	17,317	2266
Discarded IPUs^b^, *n* (%)	11,915 (29.4)	11,916 (30.8)	9426 (26.4)	6872 (20.1)	5638 (16.6)	4636 (15.4)
RCC processing						
RCCs produced, (labelled), *n*	41,953	40,657	39,135	40,663	39,527	38,826
Discarded RCCs, (processing lab), %	0.58	0.41	0.41	0.45	0.35	0.33
Plasma recovery and supply						
Average volume of plasma recovered per WB donation (mL)	256.0	259.6	259.5	260.4	258.3	265.0
Total plasma units from WB donations sent to the fractionation industry, n	37,510	40,320	37,872	37,821	41,371	40,677
Total volume of plasma from WB donations sent to the fractionation industry (L)	9606	10,483	9822	9830	10,717	10,788
PC processing						
Total PCs^c^, *n*	6021	6306	6150	6422	6827	6255
Discarded PCs, %	0.21	0.55	0.66	0.64	0.45	0.76
PCs treated with Mirasol PRT, *n* (%)	939 (15.6)	3934 (62.4)	5601 (91.1)	6422 (100)	6827 (100)	6255 (100)
Expired PCs, *n*	207	77	76	42	55	15
Stock‐out events, *n*	11	1	0	0	0	0
PC supply to hospitals^d^						
Total PCs sent to hospitals, *n*	5549	6112	5916	6405	6697	6392
Average remaining PC shelf‐life upon delivery to the hospitals^e^, (days)	2.3	2.5	2.8	3.5	3.2	3.5
Expired PCs in hospitals, *n*	255	300	286	252	291	313
PC expiry rates (%)						
BTBA	3.4	1.2	1.2	0.7	0.8	0.2
Hospitals	4.6	4.9	4.8	3.9	4.3	4.9
Aragón transfusion network^f^	8.3	6.3	6.9	4.2	5.6	5.1

Abbreviations: 2C, two‐component; 3C, three‐component; IPUs, interim platelet units; *n* absolute number, % (rate) of units in each category; PC, platelet concentrate; PRT, pathogen reduction technology; RCC, red blood cell concentrate; WB, whole blood.

^a^ For each year, the new technologies and approaches implemented to improve the efficiency of blood processing are indicated in the first row. Activity data for 2014, 2016, 2017 and 2018 are not presented because they are very similar to those reported in 2015 or 2019. In addition, only total production numbers are available for the year 2013, when the buffy coat and Reveos systems co‐existed.

^b^ IPU discard rates were calculated as the percentage of discarded IPUs out of the total number of IPUs produced with 3C separation protocols.

^c^ PCs derived from apheresis and WB.

^d^ This includes split products of the processed PCs.

^e^ Without cryopreserved PCs.

^f^ The joint network of BTBA and hospitals.

The percentage of WB units separated with 2C protocols gradually increased between 2015 (4.6%) and 2023 (24.2%), with an accelerating trend from 2021 onwards (Table 1). In 2023, over 90% of WB units were separated (2C and 3C) after overnight storage, leading to improved plasma recovery [10]. This trend coincided with the implementation and routine deployment of the LHEMA software (2022), followed by changes to the decision‐making process for protocol selection and continuous improvements to the software as of February 2023. Consequently, a substantial decrease in IPU discard rates was observed between the periods before (≥26.4% between 2015 and 2020) and after implementation of the LHEMA software (16.6% in 2022 and 15.4% in 2023).

The average volume of plasma recovered per WB donation increased by up to 9 mL in 2023 as compared to 2015; this led to an increase of over 1000 L of recovered plasma in 2023 compared to 2015 (Table 1). A comparison with 2013 was not possible because Reveos had been gradually implemented during this year and only the aggregated data was available. In parallel, the number of plasmaphereses increased significantly between 2013 (n = 125) and 2023 (n = 2259), reflecting the growing demand for plasma for fractionation, which cannot be met solely by WB separation.

### PC production, discard and expiry rates and stock‐out events

Between July 2013 and December 2023, 58,908 PCs were obtained from 359,093 WB donations separated with the Reveos system. The annual production of PCs derived from WB separation increased by 13.3% between 2013 (4656 units) and 2023 (5275 units). The number of PCs collected by apheresis grew six‐fold between 2013 (166 units) and 2023 (999 units). Overall, 38,837 PCs (from WB separation and apheresis) were treated with Mirasol PRT between 2015 and 2023. No cases of septic transfusion, virus transmission or graft‐versus‐host disease were reported following transfusion of PRT‐treated PCs. In comparison, one possible case of septic transmission by a non‐PRT‐treated PC occurred in the year 2015 [10]. Mirasol‐treated PCs were transfused to all clinical indications, including paediatric patients who received 280 splits from WB separation and 873 from apheresis.

Interestingly, PC discard rates increased from 0.21% in 2015 to 0.76% in 2023 (Table 1) following the implementation of the TOMEs middleware. This middleware, by providing step‐by‐step guidance throughout the pooling process, prompts operators to verify the sealing integrity of each connection. As a result, it has identified several sealing failures that would have otherwise gone unnoticed or underreported. Moreover, this middleware ensures that the correct IPUs are pooled. In the past, before the automated recording of the pooling process, erroneous pooling of IPUs could have happened, but as with sealing failures, these might have been underreported.

On the other hand, PC expiry rates decreased from 3.4% in 2015 (i.e., when 15.6% of the PCs were PRT‐treated) to 1.2% in 2019 (i.e., when 62.4% of the PCs were PRT‐treated). Furthermore, the lowest PC expiry rate was observed in 2023 (0.2%), with universal PRT treatment of PCs and implementation of the LHEMA software (Table 1). Most of the expired PCs at the beginning of the implementation (45 out of 55 in 2022) were caused by operators not following software instructions and producing more PCs than recommended. Cryopreserved platelets were used in a few hospitals in the region: in 2023, 97 cryopreserved PCs were transfused in seven hospitals.

The annual number of PC inventory stock‐outs rapidly declined as the percentage of units treated with Mirasol PRT increased and following the implementation of the LHEMA software (Table 1). From April 2022 to December 2023, the LHEMA software anticipated eight PC shortages. BTBA was able to avoid these potential PC stock‐outs by recruiting additional donors as recommended by the LHEMA software.

### Remaining shelf‐life and expiry rates of PCs in hospitals

The average remaining shelf‐life of PCs upon delivery to the hospitals increased by 2 day between 2015 (2.3 days) and 2023 (3.5 days) with the use of PRT and LHEMA software. The largest increase was from 2.8 days in 2020 to 3.5 days in 2021, following the universal application of the Mirasol treatment to PCs and subsequent implementation of the production algorithm (Table 1). Average expiry rates at the hospitals did not change much along the observation period, although a reduction of expiry rates could be seen in a few hospitals (data not shown).

### Staff satisfaction survey

One operator did not participate in the survey. In general, operators indicated that they were satisfied with the usability aspects of the Reveos system in comparison with the semi‐automated buffy coat method they were previously using. The scores of all evaluated items ranged between 4.0 and 4.8 (Table S1).

The surveyed participants declared they were satisfied with the different technologies introduced to support their daily routine work. All evaluated parameters had a satisfaction score of 4.0 or higher (Table S1). None of the seven operators who participated in both surveys declared that they would like to switch back to semi‐automated methods (qualitative answer). Only 1 out of the 12 operators using the TOMEs middleware would accept working again without this tool (qualitative answer). According to the operators, the most important added value of any equipment or technology was the improvement in the ergonomics (meaning that they feel safer, less exposed to hazards and repetitive strain injury while working in their current workstation), followed by error minimization, optimization of blood processing and workload reduction.

## DISCUSSION

Spain is facing the dual problem of an ageing blood donor population coupled with difficulties in retaining young donors. At the same time, the demand for PCs and plasma‐derived medicinal products is growing, highlighting the need to improve efficiencies in blood banking [22]. The objective of this report was to describe the experience of BTBA with an ecosystem of automated solutions progressively implemented to contribute to the sustainability of the blood transfusion service.

The implementation of several technologies allowed process optimization by matching WB separation and IPU management by T‐Pool Select, as well as shelf‐life extension to up 7 days through PRT treatment of PCs with real‐time platelet demand estimated by a continuous oversight of trends in component usage. The latter ensures that PCs do not remain at BTBA too long, allowing them to be dispatched to hospitals with a longer remaining shelf‐life.

The integration of this technological ecosystem, combined with an increase in platelet apheresis collections and the capability to supply cryoconserved PCs to remote hospitals with low consumption, played a crucial role in maintaining a balance between production and demand. This approach effectively minimized overproduction, reduced expiry rates, decreased waste and prevented PC stock‐outs.

However, it is important to note that BTBA does not directly manage PC inventory at hospitals. As a result, the observed decrease in expiry rates could not be universally confirmed across all hospitals in the region. This observation points to potential inefficiencies in PC inventory management within some hospitals, indicating that further optimization may be needed at this level.

Additionally, the availability of frozen PC stock has proven to be a valuable local resource, particularly in mitigating supply limitations during natural or human‐induced emergencies, such as snowstorms, floods or traffic disruptions.

By limiting overproduction and maximizing recovery of valuable resources, the new ecosystem of technologies is also expected to yield major budgetary savings for BTBA, although it is difficult to precisely quantify the economic benefits. An obvious benefit was a decrease in PC expiry rates observed throughout the analysed period. Plasma, considered a strategic resource to produce medicinal products [23], may represent a significant economic income for BEs. In Europe, fresh frozen plasma is currently selling at €155/L [24]. Consequently, the increased recovery of plasma by maximizing the ratio of 2C to 3C runs, generated a lucrative opportunity for BTBA [10].

Within the ecosystem of automated solutions, information technology (IT) connectivity increases data traceability and improves process control by rapidly detecting any potential malfunction or device defect. TOMEs contributed to automating the documentation of pooling activity (data capture and management) through bidirectional communication with the blood bank's information system (E‐Delphyn data management system, GPI Iberia). Its implementation led to an observed increase in PC discard rates. This rise is attributed to the enhanced monitoring capabilities introduced by TOMEs, particularly in the pooling process, and in the sealing performance. Although this resulted in more discards, these discards reflect failures that previously went underreported. By identifying and eliminating these issues, the system ultimately improves overall efficiency and safety in the blood component production process.

Interestingly, the automation of data entry in the blood bank information system shortens the processing time and decreases the risk of errors while enabling practical information to be added to the product label (e.g., platelet count). In view of the high amount of data collected, transferred and stored, the ultimate question is how to use this information base for improved personalized transfusion medicine. A future discussion with practicing clinicians will give more insights into the perceived benefits of such information.

Finally, the surveyed operators declared that they were generally satisfied with the automated technologies incorporated in their daily routine work, thereby indicating the positive impact of the ecosystem on human resource management. The experience with these automated tools showed that they are very intuitive and easy to use, which facilitated training in a context of high staff turnover (80 Reveos operators in the BTBA over 10 years). The broad implementation of automated technologies in BEs is intended to assist skilled operators in performing their daily activities, thus allowing the staff to allocate more time to other valuable tasks such as implementing new technologies, improving processes or performing research activities.

In conclusion, BTBA activity data during the 2013–2023 period revealed that the progressive implementation of several automated technologies within a highly connected IT network improved traceability and productivity, reduced waste and PC stock‐outs and generated staff satisfaction. These new IT‐connected processes have proven instrumental in optimizing the blood supply chain while maintaining blood product quality and safety along with an affordable cost compensated by different forms of resource savings and/or income (e.g., avoidance of overproduction, increased recovery of plasma, decreased need for laboratory space, reduction of staff). In that respect, interconnected automated processes are the answer to some of the challenges currently faced by BEs to achieve the desired goal of building sustainable health systems at cost neutrality.

## CONFLICT OF INTEREST STATEMENT

A.I.P.A. received speaker's fee and grants from Terumo Blood and Cell Technologies. I.A. founded, holds shares in and works for Hemotic. M.C. is an employee of Terumo Blood and Cell Technologies. The other authors have nothing to declare.

## Supporting information


**Table S1.** Results of operator feedback surveys in 2013 and 2023.

## Data Availability

The data that support the findings of this study are available from the corresponding author upon reasonable request.
