# Supplementary material for: An ecosystem of interconnected technologies to increase efficiencies in blood establishments: The example of the Blood and Tissue Bank of Aragón, Spain
Source: Vox Sang. 2024 Oct 23;120(1):32–8. doi: 10.1111/vox.13752 (PMC11753821; doi:10.1111/vox.13752)
Supplement: Supplementary file 1 — Table S1. Results of operator feedback surveys in 2013 and 2023. [file VOX-120-32-s001.docx]

**Supplementary table.** Results of operator feedback surveys in 2013 and 2023.

| **Survey 2013** | **Score^a^** |
| --- | --- |
| **Reveos separation system (N=13)** |  |
| Ease of loading (collect bag in centrifugation cup) | 4.5 |
| Ease of loading (lines and satellite bags) | 4.5 |
| User friendliness of the device | 4.5 |
| Opening of the break-away connectors | 4.0 |
| Quality of the seals | 4.8 |
| Ease and speed of red blood cell filtration | 4.0 |
| **Survey 2023** | **Score** |
| **Reveos separation system (N=15)** |  |
| Overall satisfaction | 4.7 |
| Utility | 5.0 |
| Intuitiveness and ease of use | 5.0 |
| Reduction of workload | 4.4 |
| Reduction of processing time | 4.7 |
| Process optimization (PYI and fractionation protocols) | 5.0 |
| Reduction of blood component discard rates | 4.8 |
| **T-Pool select software (N=15)** |  |
| Overall satisfaction | 4.6 |
| Utility | 5.0 |
| Intuitiveness and ease of use | 4.7 |
| Reduction of workload | 5.0 |
| Reduction of processing time | 5.0 |
| PC standardization | 4.3 |
| Minimization of errors | 4.0 |
| **LHEMA software (N=17)** |  |
| Intuitiveness and ease of use | 4.6 |
| Fulfillment of objectives^b^ | 4.0 |
| **TOMEs middleware (N=16)** |  |
| Overall satisfaction | 4.6 |
| Utility | 4.7 |
| Intuitiveness and ease of use | 4.9 |
| Reduction of workload | 4.0 |
| Reduction of processing time | 5.0 |
| Improvement of traceability and equipment control | 5.0 |
| Minimization of errors | 5.0 |

Abbreviations: IPU, interim platelet unit; N, number of respondents; PC, platelet concentrate; PYI, platelet yield indicator; 2C, 2-component; TOMEs, Terumo Operational Medical Equipment Software.

^a^ A scale from 1 to 5 was used.

^b^ The following objectives were evaluated: optimization of blood component separation, reduction of PC expiry rates and IPU discard rates, maximization of plasma recovery with 2C protocols, and avoidance of PC stock-outs.
